# Passive leg raising test with minimally invasive monitoring: the way forward for guiding septic shock resuscitation?

**DOI:** 10.1186/s40560-017-0232-1

**Published:** 2017-06-08

**Authors:** Patrick M. Honore, Herbert D. Spapen

**Affiliations:** Intensive Care Medicine, ICU Department, Universitair Ziekenhuis Brussel, Vrije Universiteit Brussel, 101, Laarbeeklaan, 1090 Jette, Brussels Belgium

**Keywords:** Passive leg raising test, Minimally invasive monitoring, Fluid challenge, Volume loading, Fluid removal, Septic shock, Continuous renal replacement therapy

## Abstract

**Background:**

Swift and adequate fluid loading is a cornerstone of septic shock therapy. Yet, careful assessment of volume responsiveness and volume amount during the resuscitation process is a prerequisite. Both overzealous initial fluid administration and late fluid overload are harmful and may be associated with increased mortality.

**Main body:**

Static (i.e., central venous or pulmonary artery occlusion) pressure readings are erroneous for monitoring fluid resuscitation and should be abandoned. Dynamic measurements (i.e., stroke volume and pulse pressure variation) better predict fluid responsiveness than static filling pressures but the conditions necessary for these parameters to correctly evaluate preload dependency are frequently not met. The passive leg raising maneuver as a means to alter biventricular preload in combination with real-time measurement of cardiac output changes is an easy-to-use, fast, relatively unbiased, and accurate bedside test to guide fluid management and to avoid fluid overload during early septic shock treatment. Moreover, PLR may also be particularly useful to assist various treatments that trigger fluid removal during the “de-resuscitation” phase of septic shock.

**Conclusions:**

The passive leg raising maneuver in combination with real-time measurement of cardiac output changes is an easy-to-use, fast, relatively unbiased, and accurate bedside test to guide fluid management during septic shock.

## Main text

The recently published Surviving Sepsis Campaign guidelines strongly recommend ample volume resuscitation during the first day of septic shock treatment [1]. Such aggressive fluid challenge is intended to rapidly improve tissue perfusion by increasing stroke volume and thus cardiac output (CO). However, only half of the patients respond to a fluid load, its hemodynamic benefit is short-lived, and overzealous fluid administration during the first 24 h after admission was recently found to significantly increase mortality [2, 3]. The presence of hypotension and shock requires correct assessment of volume status (cardiac preload) and accurate evaluation of the response to a fluid challenge (volume responsiveness). Traditionally, the central venous and pulmonary artery occlusion pressure have been used as surrogate estimates of respectively right and left ventricular preload. Yet, a large amount of studies have demonstrated that these static “filling” pressures poorly predict fluid responsiveness and are unable to guide fluid resuscitation [4]. Pursuing higher filling pressures may even have deleterious effects on kidney function by enhancing venous congestion and blocking venous outflow [5].

At the beginning of this millennium, several authors proposed to use physiologic heart-lung interactions during positive pressure ventilation as more reliable predictors of fluid responsiveness in septic shock [6]. Variations in both pulse pressure (PPV) and stroke volume (SVV) were found to be better tools to assess volume responsiveness than static pressure monitoring [7]. However, enthusiasm became curbed when monitoring of these dynamic variables failed to obtain optimal volume loading in both anesthetized [8] and critically ill patients [9]. Moreover, PPV and SVV measurements are reliable only under strict conditions and become significantly biased or impossible to interpret in spontaneously breathing patients, low tidal volume ventilation, and the presence of cardiac arrhythmias [10]. By far, the most simple and performant method to determine fluid responsiveness at the bedside is the passive leg raising (PLR) test. Lifting the patient’s legs from zero to about 45° produces a rapid, temporary, and reversible increase in ventricular preload by increasing venous return from the lower extremities. PLR thus perfectly mimics fluid administration without having to give exogenous fluids and is most effective when performed in association with minimally invasive monitoring for real-time tracking of changes in CO [11]. In a recent issue of the *Journal of Intensive Care*, Krige et al. prospectively investigated the use of a novel generation Vigileo FloTrac™ system during a PLR maneuver in medico-surgical patients with vasopressor-dependent circulatory shock [12]. The study population almost entirely consisted of patients with septic shock or severe systemic inflammation (pancreatitis, intestinal ischemia). These investigators found that the recorded changes in CO, using a ≥9% increase as cut point, predicted preload dependency with good sensitivity and specificity. This study definitely contributes to the growing literature on easy-to-use bedside monitoring of early volume resuscitation. However, evident limitations must be noticed. The sample size is small, all patients were mechanically ventilated from which only 20% were on spontaneous breathing, and the choice of bolus dose and cut-off value to define fluid responders remains arbitrary.

Through the years, awareness has risen that after initial aggressive fluid resuscitation the focus should shift towards obtaining a net and even negative fluid balance [13]. Late liberal fluid administration indeed is associated with increased morbidity and mortality [14]. This is even more relevant in septic shock where excessive volume expansion in association with release of pro-inflammatory and vasoactive peptides provokes significant damage to the endothelial glycocalyx [15]. As a result, intravascular fluid (including most of the resuscitation liquid!) will pass into the interstitial space and cause deleterious tissue edema. Eliminating excess fluid was associated with better survival in patients with acute respiratory distress syndrome [16] and septic shock [17]. However, methods to achieve this goal (e.g., intermittent hemodialysis, continuous renal replacement therapy, high-dose diuretics, and osmotic “tricks” such as rapid infusion of highly concentrated albumin or hypertonic saline, either alone or in combination) [18–20] have not been standardized. Moreover, too much fluid removal could impair CO, cause unwarranted bouts of hypotension, or impair renal function [21, 22]. Assuring the delicate volume equilibrium during the post-resuscitation phase necessitates accurate and reproducible monitoring. Dynamic measurements may not be appropriate in this setting. Patients either will be rapidly freed from sedation and started on spontaneous (supported) breathing or develop organ failure necessitating specific ventilator or extracorporeal support. In addition, volume shifting is a possible trigger among others for cardiac dysrhythmias [23]. PLR testing may be particularly useful in this situation. Monnet et al. recently observed in a predominantly septic shock population that preload dependence as assessed by a positive PLR test (i.e., a >9% increase in baseline cardiac index) predicted hemodynamic intolerance of dialysis-induced fluid removal [24]. Taken together, the studies of Krige et al. [12] and Monnet et al. [24] definitely set the pace for a more efficient fluid management in patients with septic shock. Any approach to volume handling in septic shock, however, remains an intricate endeavor and should be individualized. During resuscitation, CO is not only influenced by fluid administration but also by changes in myocardial contractility and compliance. Comorbid conditions such as pre-existing kidney injury and severely impaired lung function may determine both volume and speed of fluid resuscitation. Finally, the impact of ongoing inflammation and concomitant endothelial damage on fluid shifts remains largely unforeseeable.

## Conclusions

In conclusion, PLR testing in combination with more sophisticated real-time hemodynamic monitoring actually seems to be the most appropriate method to guide a bedside “do not harm” volume resuscitation strategy in septic shock. Future studies should assess the PLR maneuver using calibrated intermittent (e.g., echocardiography) or continuous (e.g., Pulse Contour Cardiac Output) monitoring and evaluate whether this test could become pivotal in managing fluid removal during the “de-resuscitation” phase of septic shock.

## Abbreviations

CO: Cardiac output
PLR: Passive leg raising
PVV: Pulse pressure variation
SVV: Stroke volume variation
